# Embryogenesis of esophageal atresia: Is localized vascular accident a factor?

**DOI:** 10.4103/0971-9261.71745

**Published:** 2010

**Authors:** Ralf-Bodo Tröbs, Ingo Stricker

**Affiliations:** Department of Pediatric Surgery, Catholic Foundation Marien Hospital Herne, D-44627 Herne, Widumer Strasse 8, Germany; 1 Institute of Pathology, BG University Clinics Bergmannsheil, Ruhr University Bochum, D-44789 Bochum, Bürkle de la Camp – Platz 1, Germany

Sir,

We have read with great interest the paper from Dutta and Harsh.[1] The authors describe observations in a case of isolated esophageal atresia (EA) without fistula (Vogt II, Gross A). During surgery, both blind-ending esophageal segments were found to be connected by an interposed atretic cord. This cord was excised, and a primary anastomosis was established.

To explain the pathogenesis of this type of EA, the authors favored a localized vascular accident during the intrauterine period, resulting in consequences to regional microcirculation and secondary atresia in the postembryonic stage. These suggestions are based on clinical observation, and are supported by *in utero* animal experiments. In detailed anatomical studies on human cadavers, Lister (1964) showed a poorly vascularized gap between the upper and lower esophagus, which is identical to the common level of EA.[2]

As the authors have reported, histopathology of the excised cord had revealed the “presence of disorganized striated muscle groups without any lumen”.[1]

In fact, the histological nature of these remnants has not been described in detail in the literature. With this background, we can add two further observations on babies with EA type Vogt II, in which the esophageal segments of both babies were connected by an atretic cord. In our cases, the connecting cord was histologically investigated.

**Figure 1:**
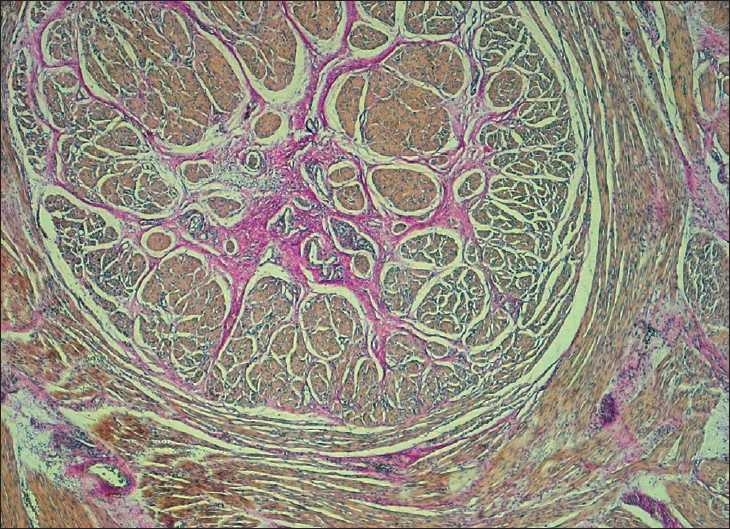
Transversal section of the atretic cord: well-organized muscle bundles separated by connective tissue (van Gieson, 25×)

**Figure 2:**
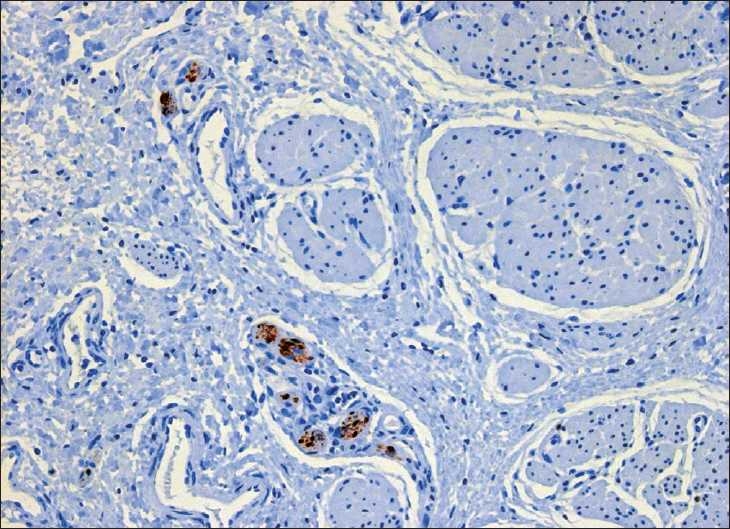
Small ganglionic cells (arrow) within the atretic part (S-100 staining counterstained with Mayer’s hematoxylin, 200×)

In the first case[3] (a female twin who was 1.6 kg after 31 weeks of gestation), the atretic cord appeared as a well-developed solid muscular structure [Figure 1]. It consisted of a circular muscle sheet next to a layer of longitudinal smooth muscle fibers separated by loose fibrous septa. Fibrous tissue with small blood vessels was present within the core. Immunostaining with S-100 antibodies revealed the presence of ganglionic cells in the cord [Figure 2]. Epithelial cells were not present.

In the second case[4] (a male who was 2.7 kg after 39 weeks), we observed a type II EA with tubular duplication of the upper esophagus. Histology of the cord revealed bundles of smooth muscles separated by connective tissue. Nerves were seen between the muscles. Immunohistochemical investigations with markers for cytokeratin (MNF 116, AE1/AE3) excluded the presence of epithelial remnants.

In conclusion, both observations provide further support for the theory of a localized vascular catastrophe. Furthermore, the composition of the atretic remnants can be characterized by three points: (1) the presence of well-orientated muscular structures, (2) the persistence of nerve bundles and ganglionic cells, and (3) the disappearance of epithelium and lumen.

In addition, the myoarchitecture found in our first case indicates the former presence of a fetal esophagus becoming atretic during the fetal period.
